# Honey Bee (*Apis mellifera*) Queen Reproductive Potential Affects Queen Mandibular Gland Pheromone Composition and Worker Retinue Response

**DOI:** 10.1371/journal.pone.0156027

**Published:** 2016-06-09

**Authors:** Juliana Rangel, Katalin Böröczky, Coby Schal, David R. Tarpy

**Affiliations:** 1 Department of Entomology, North Carolina State University, Campus Box 7613, Raleigh, NC, 27695, United States of America; 2 W.M. Keck Center for Behavioral Biology, North Carolina State University, Campus Box 7613, Raleigh, NC, 27695, United States of America; Universidade de São Paulo, Faculdade de Filosofia Ciências e Letras de Ribeirão Preto, BRAZIL

## Abstract

Reproductive division of labor is one of the defining traits of honey bees (*Apis mellifera*), with non-reproductive tasks being performed by workers while a single queen normally monopolizes reproduction. The decentralized organization of a honey bee colony is maintained in large part by a bouquet of queen-produced pheromones, the distribution of which is facilitated by contact among workers throughout the hive. Previous studies have shown that the developmental fate of honey bee queens is highly plastic, with queens raised from younger worker larvae exhibiting higher measures of reproductive potential compared to queens raised from older worker larvae. We investigated differences in the chemical composition of the mandibular glands and attractiveness to workers of “high-quality” queens (i.e., raised from first instar worker larvae; more queen-like) and “low-quality” queens (i.e., raised from third instar worker larvae; more worker-like). We characterized the chemical profiles of the mandibular glands of high-quality queens and low-quality queens using GC-MS and used the worker retinue response as a measure of the attractiveness to workers of high-quality queens vs. low-quality queens. We found that queen quality affected the chemical profiles of mandibular gland contents differently across years, showing significant differences in the production of the queen mandibular pheromone (“QMP”) components HVA and 9-HDA in 2010, but no significant differences of any glandular compound in 2012. We also found that workers were significantly more attracted to high-quality queens than to low-quality queens in 2012, possibly because of increased attractiveness of their mandibular gland chemical profiles. Our results indicate that the age at which honey bee larvae enter the “queen-specific” developmental pathway influences the chemical composition of queen mandibular glands and worker behavior. However, these changes are not consistent across years, suggesting that other external factors may play important roles in modulating queen quality.

## Introduction

By definition, highly eusocial insects exhibit extreme reproductive division of labor [1], whereby reproduction is typically monopolized by one or a few queens. The rest of the non-reproductive tasks, including brood rearing, nest building, defense, and foraging, are performed by the workers [1–3]. Honey bees (*Apis mellifera*) exhibit extreme reproductive division of labor, with tens of thousands of sterile female workers performing all non-reproductive tasks for a colony. In contrast, one queen monopolizes reproduction, mating with several males (range = 10 to 28 mates depending on the subspecies) during one to several mating flights [4–6] and laying up to 1,500 eggs daily [7]. A queen’s developmental fate is highly plastic [8–11], and her reproductive physiology is greatly affected by the queen-rearing environment. Previous studies have shown that in queenless colonies, the age of larvae when chosen by workers to be raised as queens can range from the first to the third larval instars [12–13]. This plasticity in queen phenotype leads to inherent variation in the reproductive potential of mated queens, with queens that develop from third instar larvae (compared to those that develop from first instar larvae) being more “worker-like.” These queens usually exhibit lower individual fitness and head colonies with lower growth and productivity [11]. A number of studies have shown variation in the reproductive fitness of honey bee queens raised from different worker larval instars, as measured by body size, ovariole number, and the diameter, number, and viability of drone spermatozoa stored in the queen’s sperm-storing organ (spermatheca) [10–11,13–19]. In particular, queens raised from first instar worker larvae are larger in size, have higher ovariole number, and store more spermatozoa in the spermatheca than queens reared from third instar worker larvae [11,13,16,19]. Moreover, queens raised from first instar larvae mate more frequently and have higher spermatozoa counts in the spermatheca than queens raised from third instar larvae [16].

Variation in queen reproductive potential also has colony-wide effects. For example, a recent study showed that new colonies headed by queens raised from first instar larvae built significantly more worker and drone comb, and stored more honey and pollen throughout their first season, than colonies headed by queens raised from third instar larvae [11]. This increase in colony productivity in colonies headed by queens raised from first instar larvae could be partially modulated by the higher worker attractiveness toward the mandibular gland components of higher-quality queens. If this is the case, workers in these colonies could be using the queen’s pheromonal signature as an indicator of her reproductive phenotype (reviewed by [20]).

In a honey bee colony, task organization and productivity are carefully regulated by a wide array of glandular pheromones produced by the queen [20–22]. Although the complete chemical composition of queen-produced compounds has not been fully characterized [23], a blend of the main compounds produced by queen mandibular glands include (*E*)-9-oxo-2-decenoic acid (9-ODA, which contributes 80% of the total glandular secretions in mated queens), methyl 4-hydroxybenzoate (HOB), (*R*)- and (*S*)-(*E*)-9-hydroxy-2-decenoic acid (9-HDA), 4-hydroxy-3-methoxyphenylethanol (HVA), 10-hydroxy-decanoic acid (10-HDAA) and 10-hydroxy-2 (*E*)-decenoic acid (10-HDA) [24–27]. The two enantiomers of 9-HDA and the aromatic compounds, HVA and HOB, are referred collectively as the queen mandibular pheromone, or “QMP” (see [26] for a review). QMP elicits long-term physiological (“primer”) and short-term behavioral (“releaser”) responses in workers which vary depending on the genetic composition of the colony [22, 24, 28–29]. In terms of reproduction, QMP inhibits the production of new queens [30–32], suppresses the activation of worker ovaries [33–34], and serves as a sex attractant for drones during mating [35–36]. In workers, QMP stimulates pollen and nectar foraging [37–39], delays the age-of-onset for foraging [38], and lowers juvenile hormone titers [38]. Furthermore, QMP elicits a “retinue response,” a behavioral suite in which workers surround the queen, antennate, groom, and lick her, all while collecting QMP pheromone to share with other nestmates [24,28–29,40] (see Fig 8.5 in [41]). Dissemination of QMP among workers enables nestmates to recognize the queen’s presence without the need for all workers to come in direct contact with the queen [42].

The production and chemical composition of QMP and other queen mandibular gland components are regulated by the queen’s ontogeny and mating state. Previous studies have shown that the quantity and chemical composition of QMP are significantly different between virgin vs. mated queens [43], laying vs. non-laying mated queens [44], and naturally mated vs. artificially inseminated queens [45]. Furthermore, there are differences in the chemical composition of QMP between queens inseminated with semen from one drone vs. multiple drones [46], and queens inseminated with low vs. high volumes of semen or saline solution [47]. These studies not only show that the chemical composition of QMP is highly variable, but also show that workers seem to have an inherent ability to detect these subtle differences by showing differential attractiveness to the mandibular gland chemical blends produced by queens of different reproductive phenotypes.

Queen mandibular gland chemical profiles can be highly variable, but there are three ratios that serve as good measures of queen vs. worker phenotypes. First is the ratio of two isomeric compounds (10-HDA to 9-HDA), whereby the ratio is either <1 (because queens produce more 9-HDA but have trace amounts of 10-HDA), or >1 (because workers produce more 10-HDA but have trace amounts of 9-HDA). Second is the ratio of 9-ODA to 10-HDA (which changes during queen ontogeny), whereby the ratio is either ~ 1 in virgin queens, or >1 in mated queens [43]. Lastly is the ratio of the amounts of 9-ODA/(9-ODA + 10-HDA), whereby the ratio is either ~ 1 when a mandibular gland bouquet is more “queen-like,” or close to zero when it is more “worker-like” [48–50]. Given these differences in the chemical profile of queen mandibular glands, two interesting questions arise: Do queens raised from worker larvae at different developmental stages exhibit differences in the chemical composition of their mandibular glands? If so, do those differences elicit differential responses in worker retinue behavior?

In this study, we attempted to answer these questions by raising queens from first instar and third instar worker larvae, and then allowing them to mate naturally. After the queens mated, we used a cohort of these queens to (a) dissect their mandibular glands for chemical analysis of mandibular gland components, and (b) measure retinue response of workers toward each queen type. We hypothesized that the mandibular gland chemical profiles would be different between the two experimental queen types. We also expected the ratios of the amounts of 10-HDA/9-HDA, 9-ODA/10-HDA and 9-ODA/(9-ODA + 10-HDA) to be close to the approximate values expected for mated queens. Finally, we hypothesized that workers would be more attracted to queens raised from first instar larvae during worker retinue bioassays. We discuss our results in light of other studies on the effects of queen developmental plasticity on mandibular gland composition and worker behavior, and propose some reasons for why QMP and other queen mandibular gland components may serve as indicators of honey bee queen reproductive quality.

## Methods

### Study site and bees

We conducted our study at the Lake Wheeler Honey Bee Research Facility of North Carolina State University in Raleigh, North Carolina, USA (35°43' 27", -78°40'33"). All experimental queens that were raised using the grafting method (see below) came from the same source colony headed by a naturally mated “Italian” honey bee (*Apis mellifera* L.) queen, and thus were sisters to each other. All queens reared in 2010 and 2012 were grafted from worker larvae in April and May, prime months for open mating in this area of the United States (see below for details).

### Queen rearing

All experimental queens were half-sisters to each other, since they were raised from worker larvae of known age belonging to the same source colony following established apicultural protocols [16, 51]. Briefly, we removed a frame covered in eggs and young worker larvae of approximately known age (based on the relative size of the larvae) from the source colony and thumbtacked a clear acetate sheet such that it overlaid the comb. We marked on the acetate sheet the location of all cells on that side of the frame that contained newly hatched worker larvae (approximately < 8 hours old based on larval size). We then physically transferred (“grafted”) larvae from marked cells into plastic queen-rearing cups and placed them into a queenless “cell builder” colony of bees with a high population of nurse workers ready to rear new queens [51]. Two days later, we repeated the procedure by locating a population of worker larvae previously identified on the acetate sheet, and repeated the grafting procedure to produce a second group of grafted larvae that would be approximately in the third larval instar. We placed the second group of queen cups into another queenless cell builder colony (a logistical necessity, as older worker larvae are not accepted for queen rearing in a colony that is already raising queens from younger worker larvae). Once the queens developing inside the cell builders were mature and their cells were completely sealed by workers, they were transferred into individual 2-frame mating nucleus colonies. After emergence from the cell, each queen was labeled with a paint mark on the thorax, and was allowed to roam freely and mate naturally. In this way, “high-quality” queens developed from first instar worker larvae, and “low-quality” queens developed from third instar worker larvae. We reared two sets of experimental queens for this experiment in two different years: nine high-quality queens and nine low-quality queens were reared in 2010, and eight high-quality queens and nine low-quality queens were reared in 2012 (see below).

### Collection of queens and extraction of mandibular glands

About 10 days after the queens inside mating nucleus colonies mated and the first batches of eggs were laid, we verified that they were producing viable worker brood by checking that the sealed brood cells contained worker pupae. For the cohort of queens raised in 2010, once brood viability was verified for a queen, she was collected, anesthetized by chilling at -20°C for approximately 3 min until she was immobilized, and then decapitated. For the cohort of queens raised in 2012, once brood viability was checked, 8 pairs of queens were used for one to two weeks in the behavioral assay for retinue response (see below) before they were sacrificed by decapitation, as above. After decapitation, all queen heads were immediately stored in a freezer at -80°C. The heads of both cohorts of queens were thawed in 2012, when we extracted their mandibular glands for chemical analysis (see below).

### Mandibular gland extraction and derivatization

Mandibular glands of previously frozen honey bee queens were thawed and dissected out using bee Ringer solution (0.2 g KCl, 0.2 g CaCl_2_, 4 g saccharose and 9.0 g NaCl in 1 l distilled water [52]), and placed immediately in a 1-ml tapered-bottom screw cap vial containing 50 μl of a 0.4 μg/μl solution of 10-undecenoic acid in diethyl ether (internal standard solution). Samples were kept in ice during dissections and were subsequently stored at -30°C. Twenty four hours later, 5 μl of the extract was pipetted into a 300-μl glass microinsert housed in a 1-ml autosampler vial. The solvent was allowed to evaporate before the addition of 10 μl *N*-methyl-*N*-(trimethylsilyl)-trifluoroacetamide (MSTFA). The vials were kept sealed at room temperature for 20 hours. Derivatized samples were diluted with 50 μl hexane. Blank samples were prepared at the time of dissection and contained 50 μl internal standard solution. All further handling steps for blank samples were the same as for the gland extracts.

### Chemical analysis

Samples were analyzed in a 7890A gas chromatograph (GC) system (Agilent) equipped with a DB-5 (20 m x 0.180 mm x 0.40 μm) column (Agilent) and a flame ionization detector (FID). Hydrogen was used as carrier at 56 cm/s average linear velocity. Samples were injected splitless at 280°C and the split valve opened at 0.75 min. The oven was kept at 50°C for 1 min, heated to 280°C at 5°C/min, then to 320°C at 20°C/min, and kept at the final temperature for 20 min. The FID was maintained at 320°C. One μl of derivatized sample was automatically injected using a 7683B autosampler (Agilent).

Peaks were integrated in the Kovats index range of 1495–2250 for quantification and the results were corrected manually for minor peaks when necessary. For each sample, peak area values were normalized to that of the internal standard, 10-undecenoic acid. Compounds that occurred in blank samples at comparable amounts to the amounts found in gland extracts were excluded from the analysis. FID response factors of individual compounds were not determined. Relative proportions of compounds in a sample were determined by calculating the percent normalized peak area in reference to the total normalized peak area. Since the chromatographic profiles were very similar, averages were calculated for each peak across the whole data set, and the most abundant peaks (22 in total) were selected for statistical analysis.

For identification of compounds, selected samples were analyzed on an HP-5MS (30 m x 0.250 mm x 0.25 μm) column in a 6890N GC system (Agilent) coupled with a 5975 mass selective detector (MSD; Agilent). Helium was used as carrier gas at 31 cm/s average linear velocity. Injection and temperature settings were identical to the settings described above, except that the transfer line was maintained at 320°C. Electron impact ionization with default settings was used for the MSD. Ions were detected in scan mode in the mass/charge range of 33–650 amu at a scan rate of 1.23 scans/s. Chemical identity of queen mandibular gland components was deduced based on a compound’s similarity to the mass spectra (≥ 90%) found in the reference library (Wiley 7th/NIST 05), the match of molecular weight, and the mass of characteristic fragments [53–54]. Calculated retention indices on the DB-5 column were compared to literature values [53–54], and regularities were considered within an analogous series with different chain length. The position of the double bond was not confirmed and chirality was not determined for any of the components.

### Retinue response assay

Queens raised in 2012 were used for retinue response assay using colonies housed in observation hives. To do this, we created four medium-sized colonies from four larger colonies that lived in a nearby yard, all headed by Italian queens (*Apis mellifera* L.). On 18 May 2012, we installed each of the four colonies in a three-frame observation colony as described previously [3]. The chosen frames for each observation hive contained roughly 6,000–8,000 workers, and were filled with sealed brood, honey, and pollen, to simulate the conditions present in a natural honey bee colony [41]. Bees at the entrance had access to both sides of the frames. Approximately every week, four queens (two from each treatment) were introduced at random into four separate observation hives in cages to allow workers to acclimate to the queens’ odors. After two days, the queens were released from the cages and allowed to roam freely and get accepted by workers. The retinue response assay was conducted at least one day after a queen’s release from her cage. Workers in the observation colonies were used in subsequent trials and were always unrelated to the experimental queens. The type of experimental queen that each observation hive received in subsequent trials was switched at random.

Worker retinue response to each pair of experimental queens was measured by instantaneous (point) sampling. To do this, we counted the number of workers in the queen’s retinue (i.e., number of workers antennating, licking, and surrounding the queen) once per minute for 5 minutes, and repeated this procedure each hour for five to eight hours each day, as described previously [46]. Similar instantaneous sampling was conducted daily for one to two weeks per observation hive. Once all the retinue response counts were obtained from a given queen, she was removed from the observation hive and sacrificed by decapitation as explained above. The observation hives (with the same worker force) subsequently received new pairs of experimental queens from which retinue response measurements were taken two to three days after the new caged queens were introduced. In total, we collected retinue response counts from 8 pairs of high-quality and low-quality queens, which is reported as the average value of retinue size by queen type ± s.e.m.

### Statistical analysis

Because two different cohorts of experimental honey bee queens were raised in two different years (2010 and 2012), we first analyzed the differences in the chemical composition of queen mandibular gland extracts separately by year. To do this, we performed two-way nonparametric Wilcoxon tests (because of unequal variances of the data sets) to examine any differences in the relative abundance of the 22 compounds identified from queen mandibular gland extracts based on queen grafting age. Tests were run separately for queens raised in 2010 and 2012. Because we performed several *t*-tests simultaneously on the same data set, we conducted a Bonferroni correction on the statistical values obtained each year using the total number of independent *t*-tests performed each year (α’ = α/*k*, where *k* = number of independent tests performed each year) [55].

We also used two-way nonparametric Wilcoxon tests (because of unequal variances of the data sets) to compare the ratios of 10-HDA to 9-HDA, 9-ODA to 10-HDA, and 9-ODA to 9-ODA + 10-HDA between high- and low-quality queens, which have previously been shown to differentiate queens and workers [43,48–50]. We then conducted a principal component analysis to explore differences in overall chemical profiles related to queen grafting age, based on the relative proportions of all the compounds identified from queen mandibular gland extracts across both years. The first two principal components were then analyzed by two-way ANOVA using year and queen treatment as independent variables.

Furthermore, we performed a matched-pairs *t*-test to uncover any statistical effect of queen grafting age on the mean number of workers in a queen’s retinue in observation colonies headed by high-quality or low-quality queens. We set the level of significance of the matched-pairs *t*-tests at α = 0.05. All statistical tests were performed using the statistical software package JMP Pro 10.0.0 (SAS Institute Inc., Cary, NC).

## Results

### Chemical composition of queen mandibular gland extracts

We recovered significantly more chemical material from queen mandibular gland extracts in 2012 compared to 2010 (*t* = -2.830, *P* = 0.009). The chemical analysis of mandibular gland extracts obtained from honey bee queens raised from first instar worker larvae or third instar worker larvae yielded a total of 58 different compounds that were detected in all the extracts but not in blanks. Of these 58 compounds, the 22 most abundant ones were selected for statistical analysis. We chemically identified 19 of the 22 compounds analyzed, while the remaining 3 compounds remained unidentified (Table 1). Seven of the identified compounds are known components of queen mandibular glands: HOB, 8-HOAA, 9-ODA, HVA, 9-HDA, 10-HDAA, and 10-HDA [23]. The two major components found in highest abundance were 9-ODA and 9-HDA, as expected for *A*. *mellifera* queens [43].

**Table 1:** Compounds identified using GC-MS from mandibular gland extracts of honey bee queens that were either raised from first-instar worker larvae (i.e., "high-quality" queens) or third-instar worker larvae (i.e., "low-quality" queens) in 2010 and 2012. The relative amount (in %) of each compound is given for each treatment group (mean ± s.e.m.). Kovats index values were calculated for the *N*-methyl-*N*-(trimethylsilyl)-trifluoroacetamide (MSTFA) derivatives obtained from the GC retention times. Differences in relative amounts of compounds between high-quality queens and low-quality queens were analyzed with two-tailed non-parametric Wilcoxon tests because of unequal variances. All tests were performed separately for queens raised in 2010 and queens raised in 2012 (see Methods for details).

Peak no.	Queen mandibular gland component**	Acronym	Kovats Index	Relative amount of each compound (%)*											
				Queens raised in 2010						Queens raised in 2012					
				High-quality queens (*n* = 9)		Low-quality queens (*n* = 9)		Wilcoxon test***		High-quality queens (*n* = 8)		Low-quality queens (*n* = 9)		Wilcoxon test	
				mean	s.e.m.	mean	s.e.m.	χ^2^	*P* value****	mean	s.e.m.	mean	s.e.m.	χ^2^	*P* value
1	methyl 4-hydroxybenzoate	HOB	1498	2.78	0.45	4.95	0.88	3.60	**0.05**	5.68	1.06	6.93	1.65	0.59	0.44
2	7-hydroxyoctanoic acid		1554	0.97	0.11	0.84	0.11	0.44	0.51	0.57	0.07	0.47	0.09	1.13	0.29
3	8-hydroxyoctanoic acid	8-HOAA	1626	9.23	0.87	8.27	0.93	0.38	0.54	10.25	0.83	10.43	1.14	0.01	0.92
4	4-hydroxybenzoic acid		1635	0.42	0.07	0.40	0.03	0.16	0.69	0.47	0.11	0.39	0.06	0.00	0.96
5	8-hydroxy-(*E*)-2-octenoic acid		1673	0.20	0.01	0.17	0.02	1.44	0.23	0.29	0.02	0.29	0.03	0.01	0.92
6	9-oxo-(*E*)-2-decenoic acid	9-ODA	1709	40.28	1.08	39.03	1.68	0.05	0.83	42.35	1.88	35.61	2.98	2.67	0.10
7	4-hydroxy-3-methoxyphenylethanol	HVA	1720	0.28	0.03	0.48	0.04	9.59	**0.002**	1.19	0.28	1.12	0.25	0.06	0.81
8	9-hydroxydecanoic acid		1748	1.30	0.06	1.20	0.11	1.22	0.27	0.98	0.06	1.04	0.09	0.19	0.66
9	8-hydroxy-(*E*)-2-decenoic acid		1783	0.57	0.02	0.39	0.07	6.14	**0.01**	0.62	0.02	0.57	0.03	0.84	0.36
10	9-hydroxy-(*E*)-2-decenoic acid	9-HDA	1799	29.34	0.62	24.30	1.46	7.25	**0.007**	25.89	1.62	28.08	1.66	1.12	0.29
11	*unidentified compound 1*		1815	0.56	0.05	0.49	0.06	1.22	0.27	0.36	0.05	0.32	0.10	0.33	0.56
12	10-hydroxydecanoic acid	10-HDAA	1818	1.69	0.06	2.54	0.47	5.69	**0.02**	2.40	0.29	2.99	0.58	0.28	0.60
13	10-hydroxy-(*E*)-2-decenoic acid	10-HDA	1869	3.58	0.49	4.18	0.50	1.64	0.20	5.29	0.96	7.66	0.91	5.79	**0.02**
14	decanedioic acid		1901	0.24	0.02	0.38	0.04	6.37	**0.01**	0.35	0.02	0.40	0.06	0.04	0.85
15	4-hydroxy-3-methoxyphenylpropanoic acid		1908	0.57	0.04	0.70	0.05	3.28	0.07	0.57	0.07	0.69	0.08	0.08	0.77
16	x-decenedioic acid		1953	0.46	0.06	0.57	0.06	1.42	0.23	0.60	0.05	0.66	0.08	0.23	0.63
17	10-hydroxy-(*E*)-2-dodecenoic acid		1982	0.33	0.04	0.27	0.07	0.94	0.33	0.17	0.03	0.13	0.03	0.60	0.44
18	11-hydroxy-(*E*)-2-dodecenoic acid		1992	0.43	0.03	0.42	0.02	0.02	0.89	0.37	0.02	0.36	0.04	0.00	1.00
19	*unidentified compound 2*		2020	0.64	0.05	0.75	0.19	0.12	0.72	0.68	0.12	0.53	0.12	0.45	0.50
20	9-hexadecenoic acid		2029	0.64	0.06	1.15	0.41	0.01	0.93	0.08	0.02	0.12	0.05	0.01	0.92
21	*unidentified compound 3*		2062	0.86	0.13	0.65	0.08	0.50	0.48	0.39	0.04	0.39	0.05	0.02	0.88
22	9-octadecenoic acid		2221	4.63	0.88	7.86	2.26	1.03	0.31	0.45	0.08	0.81	0.28	0.06	0.81
	Total relative amount of all compounds analyzed (%)			100		100				100		100			
	Total relative amount of identified compounds (%)			97.9		98.1				98.6		98.8			
	Total relative amount of unidentified compounds (%)			2.1		1.9				1.4		1.2			
	Total relative amount (%) of QMP compounds (sum of 1, 6, 7, 10)			72.7		68.8				75.1		71.7			
	Ratio of 10-HDA to 9-HDA			0.12		0.17		3.01	0.08	0.20		0.27		2.39	0.12
	Ratio of 9-ODA to 10-HDA			11.25		9.34		1.22	0.27	8.01		4.65		6.26	**0.01**
	Ratio of 9-ODA to 9-ODA + 10-HDA			0.92		0.90		0.92	0.34	0.89		0.82		6.31	**0.01**

* Calculated relative to the internal standard without regard to differential FID response factors.

** Chemical identity of queen mandibular gland components was deduced based on similarity to library mass spectra (≤ 90%), match of molecular weight as well as mass of characteristic fragments (see [44,45]). Calculated retention indeces on the DB-5 column were compared to literature values [43,46] and regularities were considered within an analogous series with different chain length.

*** All statistical comparisons are with non-parametric Wilcoxon tests assuming unequal variance. Ratios of 10-HDA to 9-HDA, 9-ODA to 10-HDA, and 9-ODA to 9-ODA + 10-HDA were performed to determine relative queen quality following similar analyses done previously [40, 49–51].

**** Statistically significant differences in mean absolute amounts (*P* ≤ 0.05; in bold) were re-analyzed using a Bonferroni correction for *k* = 22 different statistical tests performed each year. Therefore, the Bonferroni-corrected α' was = (0.05 / 22) = 0.002.

For queens raised in 2010, we found a few interesting differences in the relative amounts of five compounds between queen types. Mandibular gland extracts from high-quality queens contained higher concentrations of 9-HDA and 8-hydroxy-(*E*)-2-decenoic acid. Conversely, extracts from low-quality queens contained statistically higher concentrations of HOB, HVA, 10-HDAA, and decanedioic acid (Table 1). However, because we performed a total of 22 *t*-tests simultaneously on the same data set for each year, the Bonferroni-corrected α’ value (*k* = 22) was 0.002, and thus we only accepted as statistically significant those differences in relative amounts between high- and low-quality queens when α’ was < 0.002 [55]. Therefore, only the differences in HVA and 9-HDA were statistically significant in 2010. We did not observe similar differences in these two compounds from queens raised in 2012, however.

In 2012, we only found one significant difference in the relative amount of mandibular gland compounds (*P* = 0.02), with low-quality queen mandibular glands exhibiting significantly more 10-HDA compared to those of high-quality queens (Table 1), but this value was insignificant after performing a Bonferroni correction. We did not observe similar differences in 10-HDA concentrations from queens raised in 2010, however. We found no other statistical differences in the chemical composition of mandibular gland extracts between queens in 2010 or 2012. Our results indicate that the chemical composition of queen mandibular gland contents from high-quality and low-quality queens varied greatly and was not consistent across treatment years.

We also used three ratios of the absolute and relative quantities of mandibular gland compounds as indicators of queen quality, all of which showed trends of greater “queenliness” in high-quality queens. The first ratio, where a low ratio 10-HDA to 9-HDA is indicative of queens, ranged between 0.12 and 0.27 in both years for relative amounts of the compounds, although these values were not statistically significant between high- and low-quality queens in either 2010 or 2012 (Table 1, S1 Table). The second indicator, whereby a ratio of 9-ODA to 10-HDA increases after queens mate and become fertile, was >1, ranging between 4.65 and 11.25 in both years for relative amounts of compounds. This ratio was significantly higher in high-quality queens in 2012 (*P* = 0.01), although it was not significantly different, albeit numerically higher, for high-quality queens in 2010 (Table 1). Finally, the third indicator, whereby a ratio of 9-ODA to 9-ODA + 10-HDA should be close to 1 for queen-like females, ranged from 0.82 to 0.92 across years for relative amounts of compounds. This ratio was significantly higher in high-quality queens in 2012 (*P* = 0.01), although it was not significantly different, albeit higher, for high-quality queens in 2010 (Table 1). All of these trends were similar when considering the absolute amount of each compound (S1 Table).

**Fig 1:**
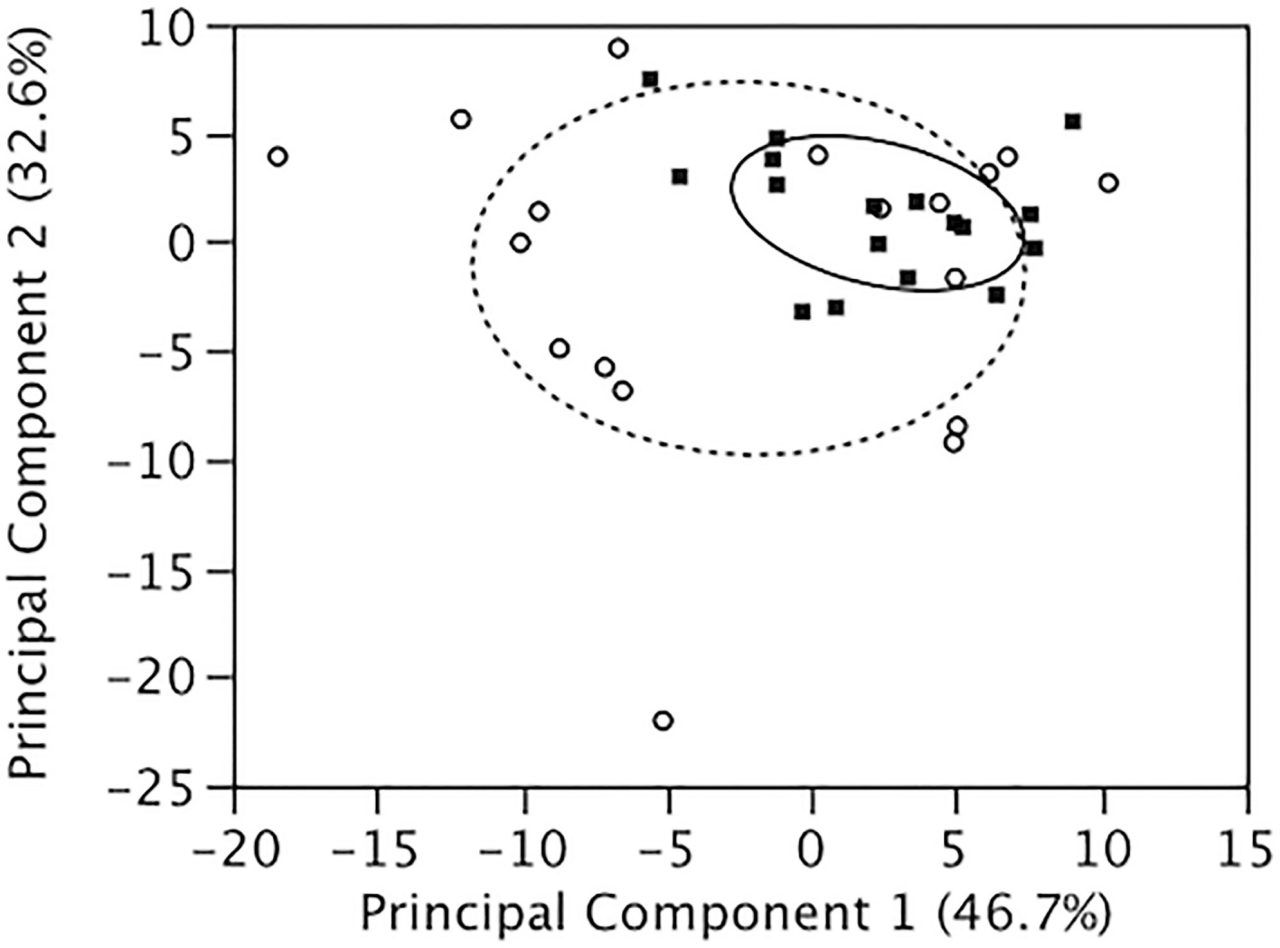
Queen grafting age significantly altered the chemical profile of mandibular gland contents. The chemical composition of mandibular gland extracts of honey bee queens raised from either first instar worker larvae (i.e., “high-quality” queens) or third instar worker larvae (i.e., “low-quality” queens) were analyzed using gas chromatography and mass spectrometry. Principal component analysis of the mandibular extracts from both queen types was done based on the relative proportion of each compound. The first principal component (PC1) explained 46.7% of the variation in mandibular gland composition. The second principal component (PC2) explained an additional 32.6% of the variation. There was a significant difference in the two-dimensional composite measure between high-quality queens (black squares) and low-quality queens (open circles). Solid and dashed ellipses signify 50% confidence intervals for PC1 and PC2 for high-quality queens and low-quality queens, respectively.

We then used principal components analysis (PCA) to analyze the 22 queen mandibular gland components that met the analysis criteria (see above) simultaneously across both years as a composite of queen reproductive quality (see [16] [46]). As analyzed by two-way ANOVA, PC1 explained 46.7% of the variance and showed a significant treatment effect (i.e., queen grafting age: F_1,31_ = 4.15, *P* = 0.05) but not an effect of year (F_1,31_ = 0.74, *P* = 0.40) or their interaction (F_1,31_ = 1.62, *P* = 0.21). PC2 explained an additional 32.6% of the variance and showed a significant year effect (F_1,31_ = 6.09, *P* < 0.05) and a treatment by year interaction (F_1,31_ = 4.39, *P* < 0.05), but no treatment effect (F_1,31_ = 2.27, *P* = 0.14). Thus principal component analysis showed that grafting age significantly affected the overall profile of the mandibular gland chemical profile in queens (Fig 1).

### Retinue response bioassays

**Fig 2:**
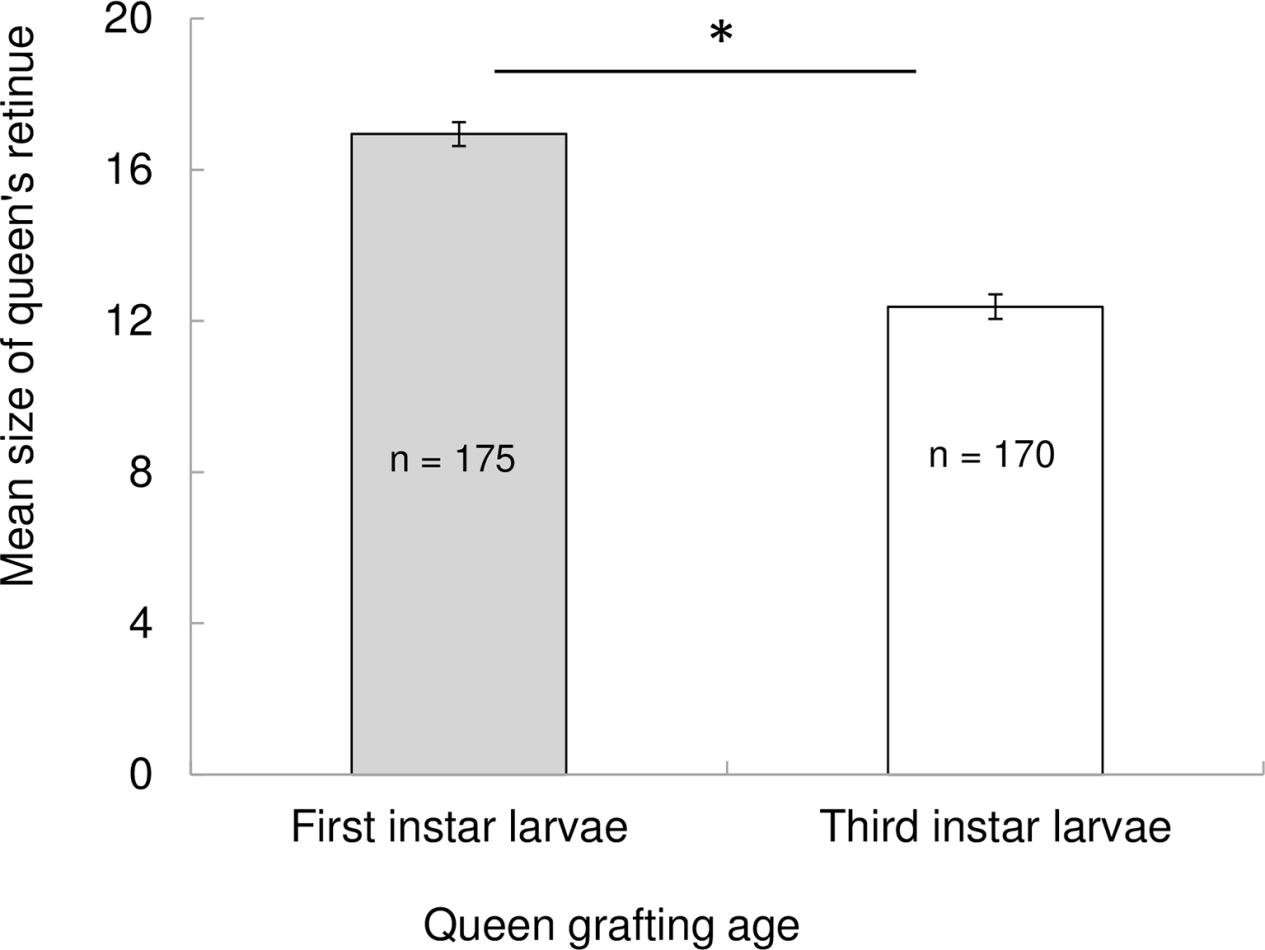
Levels of worker attraction to honey bee queens varied during retinue response bioassays depending on the age at which a worker larvae are chosen to be raised as queen (i.e., "grafting age," see Methods for details). Worker retinue size was significantly higher when observation colonies were headed by queens raised from first instar worker larvae (i.e., "high-quality" queens) compared to those headed by queens raised from third instar worker larvae (i.e., "low-quality" queens). The total number of instantaneous sampling points for each queen type (n) is denoted within each bar. Retinue size across both treatments was compared with a matched-pair t-test (* *P* < 0.0001).

Retinue response bioassays were conducted in 2012. The number of workers licking, antennating, and grooming queens introduced in observation hives was significantly higher when queens were raised from first instar worker larvae (average retinue size = 17) than when they were raised from third instar worker larvae (average retinue size = 12). High-quality queens attracted a larger number of workers in their retinue compared to low-quality queens (*t*-ratio = 11.03, d.f. = 168, *P* < 0.0001, Fig 2). Thus, queen grafting age clearly affected the retinue response of workers, with queens raised from younger worker larvae eliciting a larger retinue response than queens raised from older worker larvae.

## Discussion

In this study, we investigated whether honey bee queen reproductive potential affects specific aspects of queen physiology and worker behavior. In particular, we explored whether the age at which a female larva enters the queen developmental pathway (i.e., the “grafting age”) altered the chemical composition of the resulting queen’s mandibular glands, and whether queens raised from younger worker larvae elicited a stronger worker retinue response compared to queens raised from older worker larvae.

For the 22 queen mandibular gland compounds analyzed in this study, the mandibular gland extracts from queens raised in 2010 differed from those raised in 2012. Notably for the latter set of queens (for which we have behavioral data of queen attractiveness to workers), there was a difference in the relative concentration of only one mandibular gland component, with low-quality queens showing higher relative amounts of 10-HDA compared to high-quality queens. Perhaps the inconsistent differences in relative concentrations of mandibular gland components between the two years can be attributed to environmental factors such as the year and season when we performed the queen-rearing protocols, the time after mating when the queens were collected for analysis, or the mating frequency of low- vs. high-quality queens across both years (not measured in this study). Furthermore, principal component analysis showed that queen grafting age affected the overall chemical profile of queen mandibular glands, irrespective of the individual chemical profile differences observed between years.

Our results are consistent with those of Plettner et al. [43], who after comparing the mandibular gland chemical composition of 6-day-old virgin queens to that of mated queens 1 year post-laying, found that all but one mandibular gland components were significantly higher in the 1-year-old mated queens, where 10-HDA was produced in significantly higher levels by the 6-day-old virgin queens. Because 10-HDA is found in higher concentration in workers, whereas 9-HDA is found in higher concentration in queens and is a major QMP compound, a low ratio of 10-HDA to 9-HDA readily differentiates queens from workers [43]. Our results showed that in both years this ratio trended toward lower values in higher-quality queens, suggesting that it does serve as a good indicator of queen quality. Similarly, the ratio of 9-ODA to 10-HDA, which increases with queen age and mating status [43], also trended in the same direction in our high-quality queens. Finally, the ratio of 9-ODA to 9-ODA+10-HDA, which is an indication of “queenliness” [48–50] also trended in the right direction for high-quality queens.

Previous studies have also shown that the amounts of QMP and other mandibular gland compounds increase within weeks after queen emergence, with older queens typically exhibiting more complex and abundant mandibular gland chemical bouquets ([43,56–59], see also remarks in Table 1 and S1 Table). Likewise, our study joins a body of work showing that mating status (i.e., virgin, single-drone inseminated, multiple-drone inseminated, and naturally mated queens) also has a big influence on mandibular gland chemical composition [43,46,56,59].

The differences we observed in the chemical composition of queen mandibular glands based on grafting age need to be interpreted with caution for several reasons. First, the total amounts of volatiles obtained from experimental queens (96 μg in 2010 and 223 μg in 2012) were relatively low compared to previous studies, which have reported considerably higher values [57,58]. Furthermore, Kocher et al. 2009 [45] and Richard et al. [46] report more than 3 times higher ratios for 9-ODA/10-HDA than in our study, which could be due to the way in which mandibular glands were processed and stored. Particularly for the 2010 samples, the storage of queens in a deep freezer may have compromised the integrity of the compounds obtained from the mandibular glands.

Another source of discrepancy between ours and other studies could have been the way in which queens were reared. The necessary use of two different cell-building colonies for the production of the experimental queens could have biased the phenotype of the queens to some degree, since we cannot eliminate the possibility that nurse bees in one cell builder were less attentive in their queen-rearing duties, thus producing queens that were fed or tended to less than the other cell builder. Given that several physiological and external factors seem to affect queen mandibular gland composition in ways that we do not fully understand, queen reproductive quality is not necessarily always reflected in a quantitative or qualitative change of all compounds in the mandibular glands. Rather, reproductive quality, as measured by grafting age (this study), time after mating [43], and mating status [46,56,59] likely affect mandibular gland composition by modulating the relative concentrations (i.e., the ratio of key compounds) and is dependent on many physiological and environmental factors that are not yet fully elucidated.

This study also found that queens raised from first-instar worker larvae elicited larger worker retinues compared to queens raised from third-instar worker larvae, indicating that queen grafting age significantly affects queen attractiveness to workers. Other studies have also shown that QMP and other queen mandibular gland components regulate physiological and behavioral responses in workers [24,30,32–34,38–39,46–47,60–65], including worker retinue response. For example, Richard et al. [46] found that queen retinue size was larger in queens inseminated with semen from one vs. multiple drones. Similarly, Kocher et al. [45] showed that caged bees were more attracted to mandibular gland extracts of naturally mated queens compared to instrumentally inseminated queens and virgin queens, while Niño et al. [47] found that retinues were larger around queens that were inseminated with high versus low volumes of semen.

Nonetheless, the queen retinue sizes found in our study (i.e., 17 workers for high-quality queens, 12 workers for low-quality queens, on average) are up to three times higher than those reported by others [46,66–67]. Perhaps the high retinue values in our study emerged from differences in methodology, as we considered a worker to be part of the queen retinue if she antennated the queen for at least two consecutive seconds, while other studies may have been more stringent on their definition of a retinue worker. However, we are unable to clearly detect these differences in methods with other studies, as previous authors did not fully articulate how they decided which workers to include in retinue analysis. Despite these discrepancies in retinue size across studies, we are confident in our sampling protocol, as we point-sampled over 150 retinue observations for each experimental queen group.

Furthermore, while our large sample size for retinue behavior makes it highly likely that our observed differences in gland composition may influence the differences in worker response, we cannot exclude that the increased attractiveness of workers toward high quality queens could be due to other factors, including cuticular lipids [68], tergal gland secretions [69], or Dufour’s gland secretions [70], as queen retinue behavior has been reported toward queens whose mandibular glands had been removed [70–73].

Regardless of these possibilities, any observed differences in queen attractiveness to workers due to grafting age could have an adaptive value. Following queen-rearing protocols similar to our study, a previous investigation on the variation of queen grafting age showed that queens raised from younger worker larvae were larger in size, stored larger numbers of spermatozoa in their spermathecae, and mated with significantly more males than queens that were raised from older worker larvae [16]. A similar study showed that newly founded colonies headed by queens raised from younger worker larvae produced significantly more worker and drone comb, stored more food, and had a larger worker population than colonies headed by queens raised from older worker larvae [11]. Thus, the age of worker larvae when initially chosen to be raised as a queen seems to not only affect queen reproductive phenotype and worker retinue behavior (as shown in the present study), but it directly influences colony overall productivity over time (as shown by [10]).

Low queen attractiveness might be one of the factors influencing the collective decision by workers to either keep their queen or to replace her with a new one (queen supersedure). Rangel et al. [11] did not find significant differences in queen supersedure rate between queens raised from either young or old worker larvae, however, suggesting that other factors of a queen’s phenotype (like the ones outlined above) might be more important precursors to queen supersedure and queen failure. Nevertheless, given that queen reproductive quality is likely a complex function of queen age, mating state, initial rearing age, insemination volume, and other external factors, it is difficult to discern the individual contribution of differences in queen grafting age on differences in queen attractiveness to workers. Therefore, further studies are needed to discern the individual contributions of biotic and abiotic factors influencing queen reproductive quality, and how queen phenotype affects the physiology and behavior of the workers at the colony level.

## Supporting Information

S1 Appendix Raw data for all statistical tests. A) Raw data for the absolute amounts (in μg) and B) Raw data for the relative amounts (in % of the total amount) of each of 22 compounds identified using GC-MS from mandibular gland extracts of honey bee queens that were raised from either first-instar worker larvae (i.e., "high-quality" queens) or third-instar worker larvae (i.e., "low-quality" queens) in 2010 and 2012. Kovats index values were calculated for the *N*-methyl-*N*-(trimethylsilyl)-trifluoroacetamide (MSTFA) derivatives obtained from the GC retention times. The column titled "Total" indicates the total amount of compounds (in μg) detected from the glands of each queen. The column titled "Total id" indicates the total amount of compounds that were positively identified using GC-MS, while the column titled "Total UN" indicates the total amount of compounds that were not identified. C) Raw data for the retinue size observed during each 5-min point sampling bout from observation colonies headed by either queens raised from first instar worker larvae (i.e., "high-quality" queens) or by queens raised from third instar worker larvae (i.e., "low-quality" queens). The total number of instantaneous sampling points for each queen type (n) is denoted within each bar.
(XLSX)

S1 Table Absolute amounts of queen mandibular gland compounds. Compounds identified using GC-MS from mandibular gland extracts of honey bee queens that were raised from either first-instar worker larvae (i.e., "high-quality" queens) or third-instar worker larvae (i.e., "low-quality" queens) in 2010 and 2012. The absolute amount (in μg) of each compound is given for each treatment group (mean ± s.e.m.). Kovats index values were calculated for the *N*-methyl-*N*-(trimethylsilyl)-trifluoroacetamide (MSTFA) derivatives obtained from the GC retention times. Differences in absolute amounts of compounds between high-quality queens and low-quality queens were analyzed with two-tailed non-parametric Wilcoxon tests because of unequal variances. Tests were performed separately for queens raised in 2010 and queens raised in 2012 (see Methods for details). * Calculated relative to the internal standard without regard to differential FID response factors. ** Chemical identity of queen mandibular gland components was deduced based on similarity to library mass spectra (≥ 90%), match of molecular weight as well as mass of characteristic fragments (see [44,45]). Calculated retention indeces on the DB-5 column were compared to literature values [43,46] and regularities were considered within an analogous series with different chain length. *** All statistical comparisons are with non-parametric Wilcoxon tests assuming unequal variance. Ratios of 10-HDA to 9-HDA, 9-ODA to 10-HDA, and 9-ODA to 9-ODA + 10-HDA were performed to determine relative queen quality following similar analyses done previously [40, 49–51]. **** Statistically significant differences in mean absolute amounts (P ≤ 0.05; in bold) were re-analyzed using a Bonferroni correction for k = 22 different statistical tests performed each year. Therefore, the Bonferroni-corrected α' was = (0.05 / 22) = 0.002.
(XLSX)
